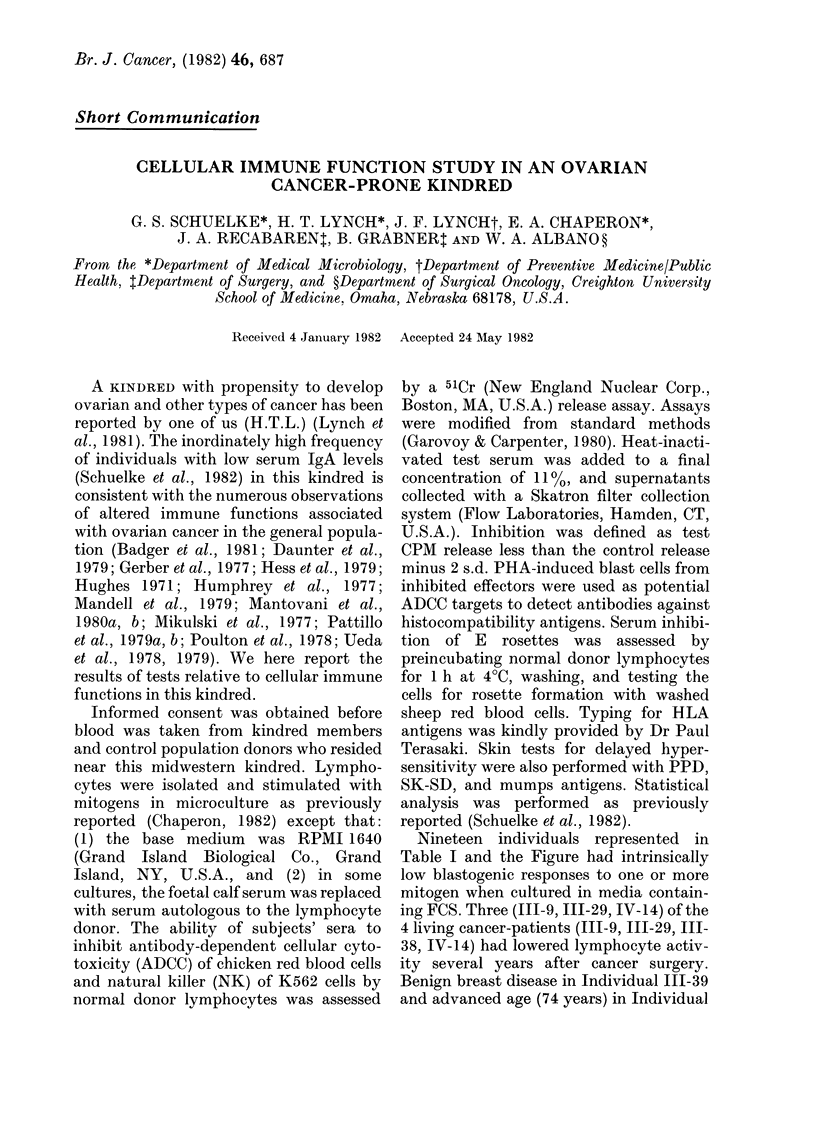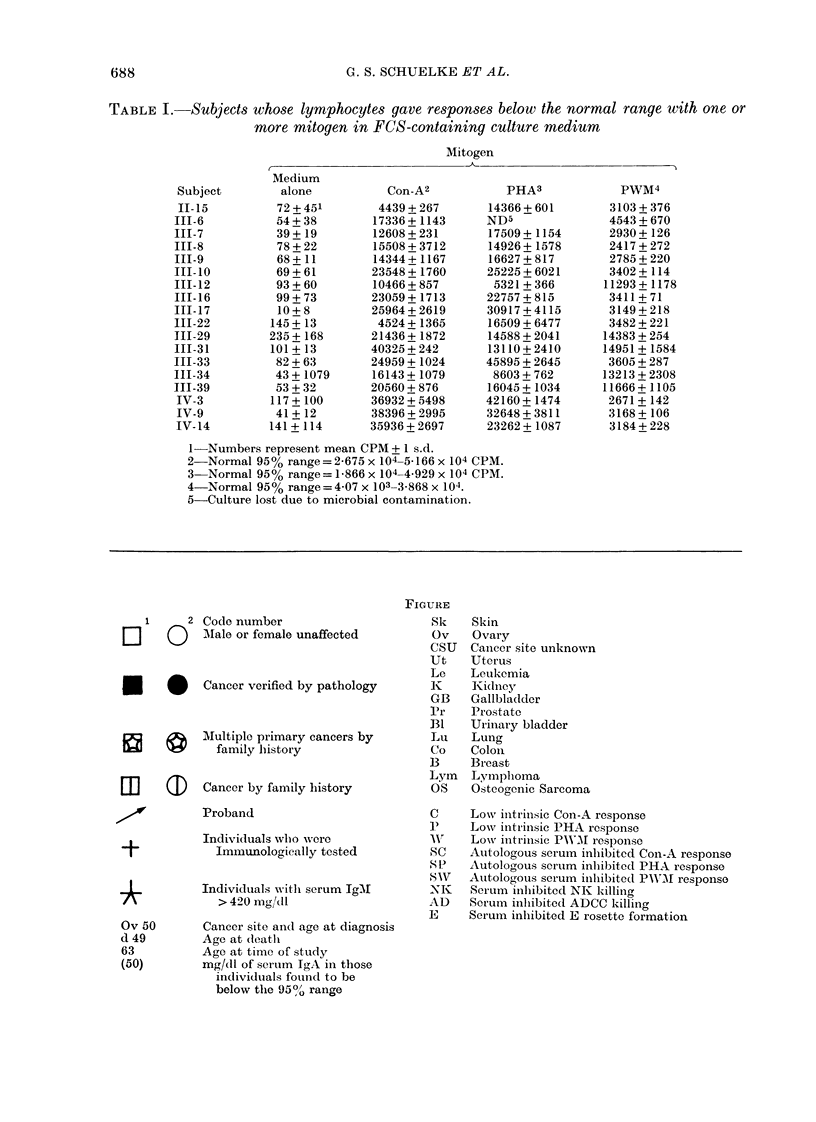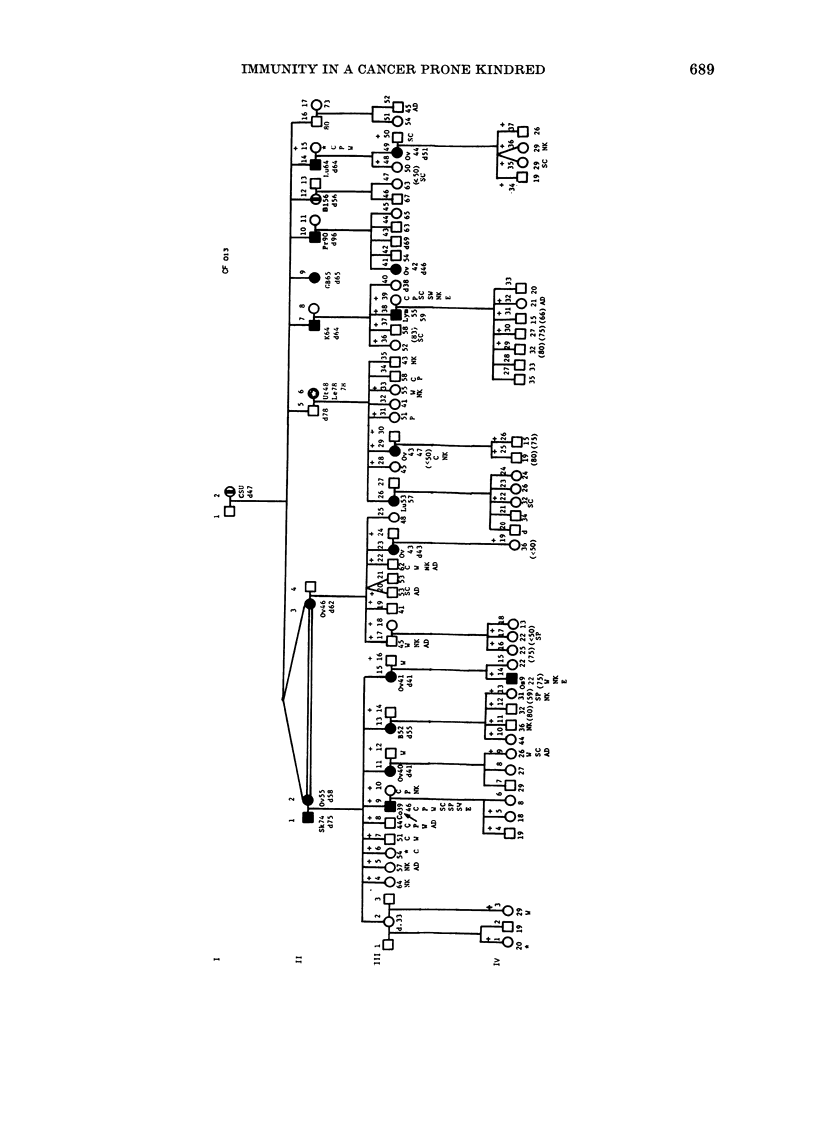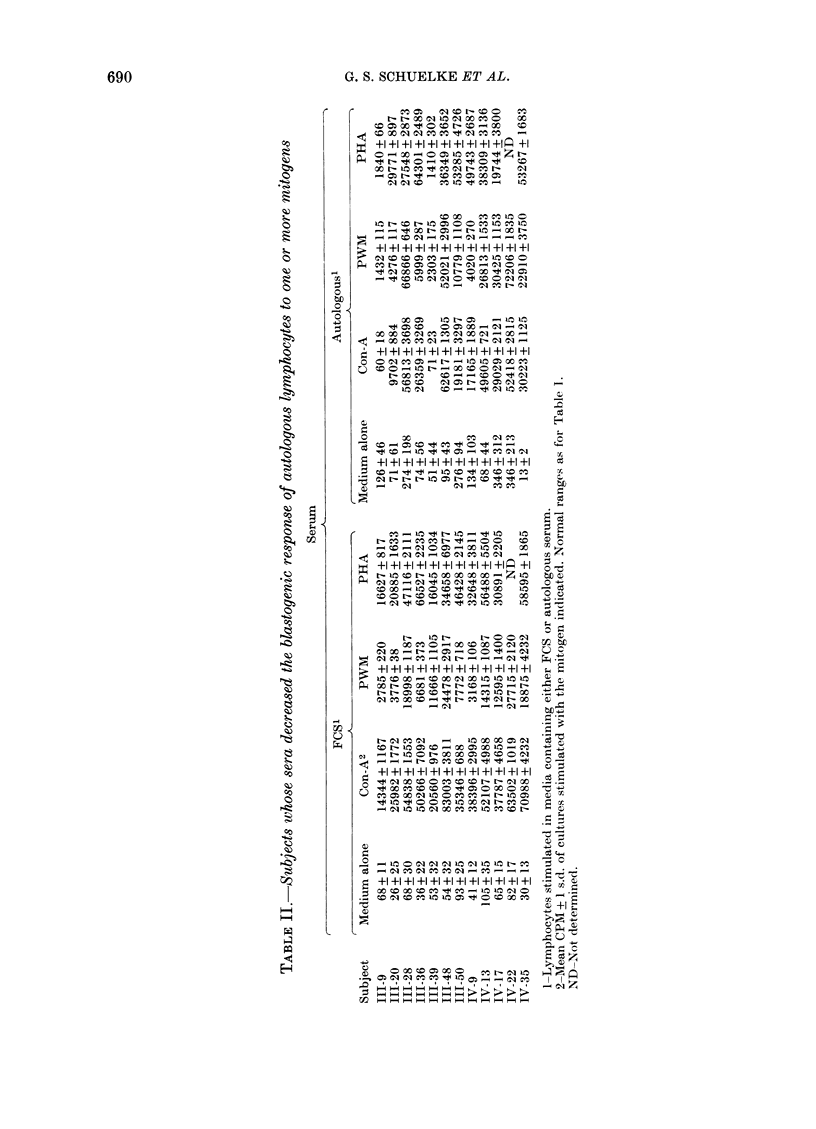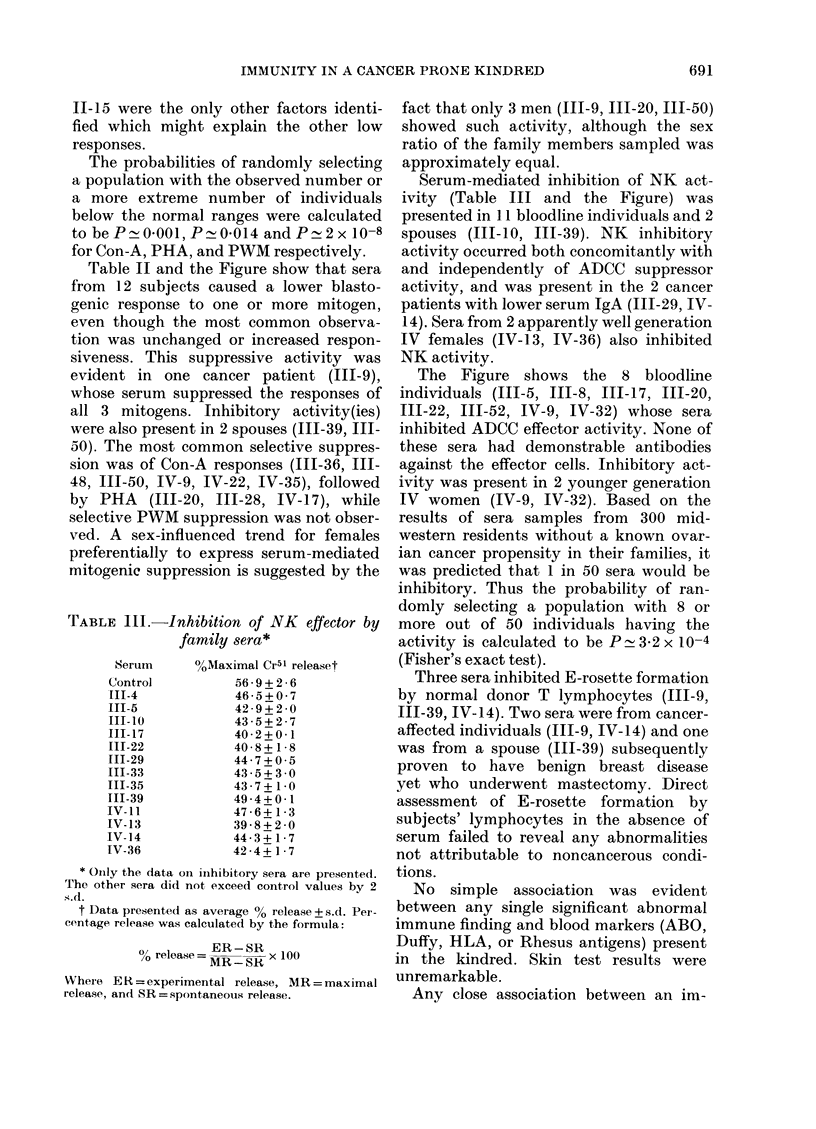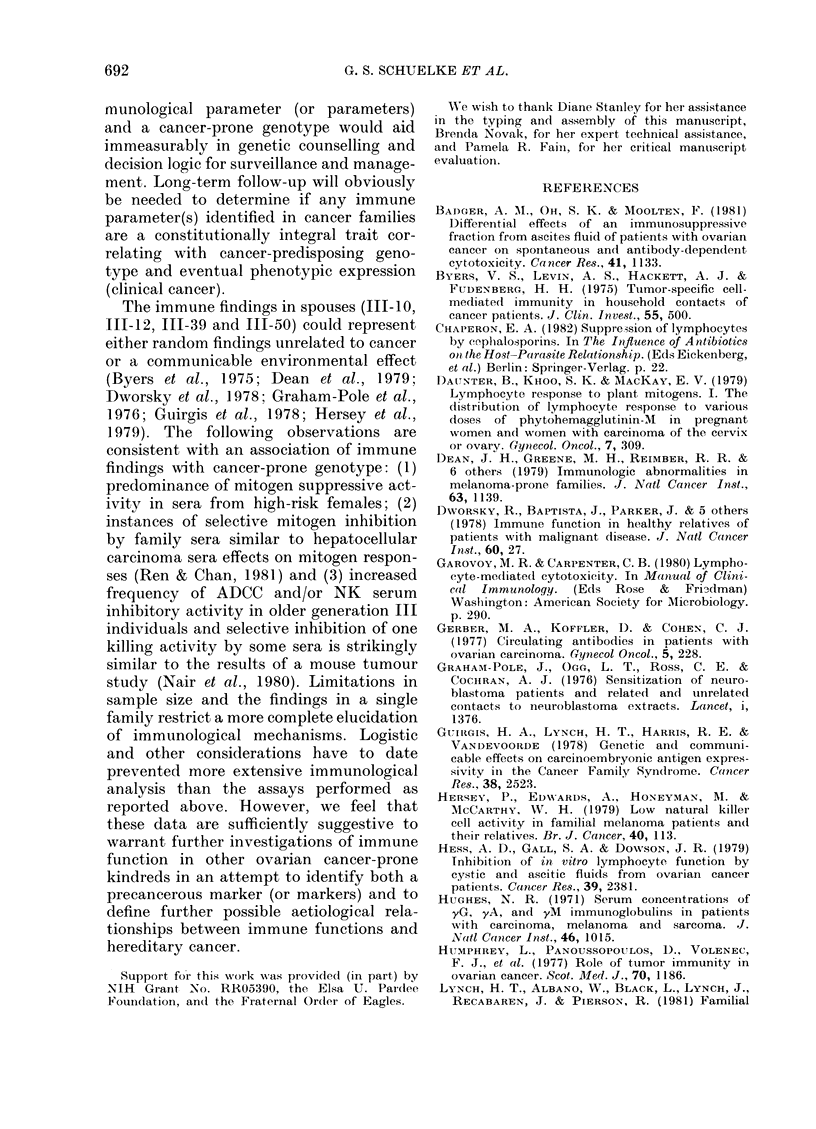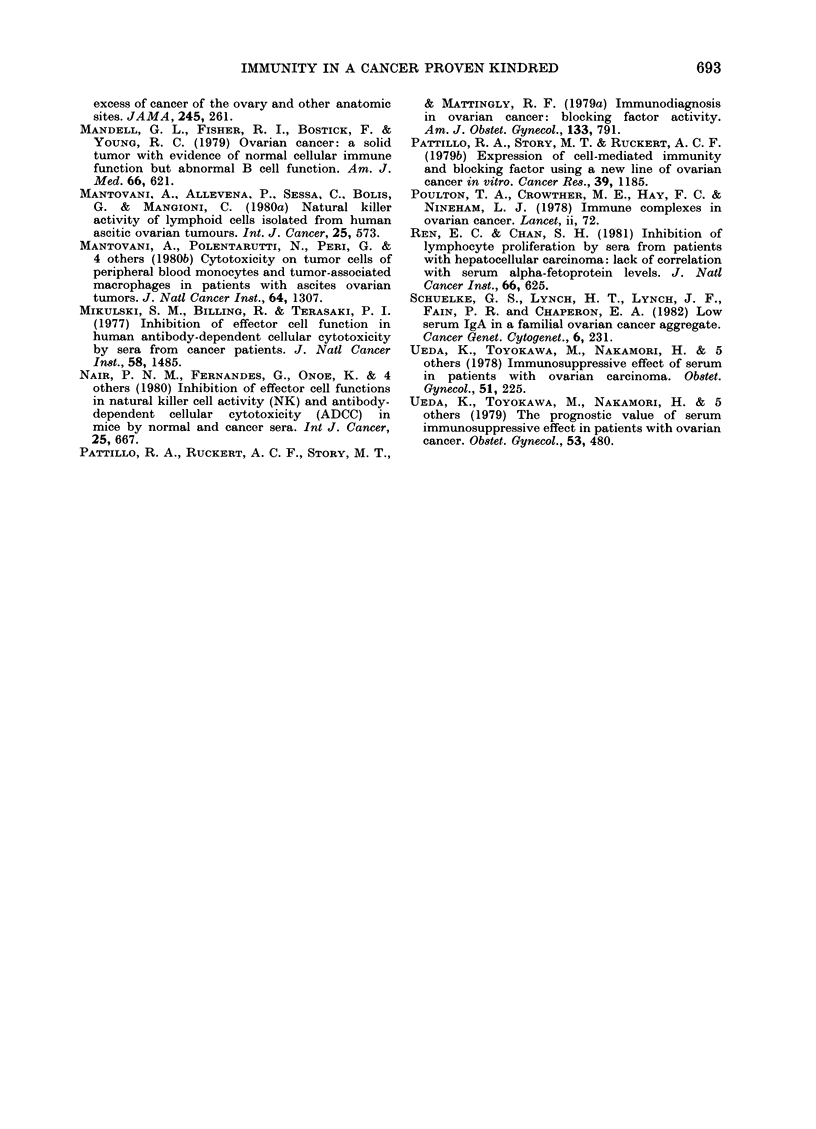# Cellular immune function study in an ovarian cancer-prone kindred.

**DOI:** 10.1038/bjc.1982.256

**Published:** 1982-10

**Authors:** G. S. Schuelke, H. T. Lynch, J. F. Lynch, E. A. Chaperon, J. A. Recabaren, B. Grabner, W. A. Albano


Br. J. Cancer, (1982) 46, 687

*Short Communication*

### CELLULAR IMMUNE FUNCTION STUDY IN AN OVARIAN CANCER-PRONE KINDRED

G. S. SCHUELKE*, H. T. LYNCH*, J. F. LYNCH†, E. A. CHAPERON*, J. A. RECABAREN‡, B. GRABNER‡ AND W. A. ALBANO§

*From the *Department of Medical Microbiology, †Department of Preventive Medicine/Public Health, ‡Department of Surgery, and §Department of Surgical Oncology, Creighton University School of Medicine, Omaha, Nebraska* 68178, *U.S.A.*

Received 4 January 1982 Accepted 24 May 1982

A KINDRED with propensity to develop ovarian and other types of cancer has been reported by one of us (H.T.L.) (Lynch *et al.*, 1981). The inordinately high frequency of individuals with low serum IgA levels (Schuelke *et al.*, 1982) in this kindred is consistent with the numerous observations of altered immune functions associated with ovarian cancer in the general population (Badger *et al.*, 1981; Daunter *et al.*, 1979; Gerber *et al.*, 1977; Hess *et al.*, 1979; Hughes 1971; Humphrey *et al.*, 1977; Mandell *et al.*, 1979; Mantovani *et al.*, 1980*a*, *b*; Mikulski *et al.*, 1977; Pattillo *et al.*, 1979*a*, *b*; Poulton *et al.*, 1978; Ueda *et al.*, 1978, 1979). We here report the results of tests relative to cellular immune functions in this kindred.

Informed consent was obtained before blood was taken from kindred members and control population donors who resided near this midwestern kindred. Lymphocytes were isolated and stimulated with mitogens in microculture as previously reported (Chaperon, 1982) except that: (1) the base medium was RPMI 1640 (Grand Island Biological Co., Grand Island, NY, U.S.A., and (2) in some cultures, the foetal calf serum was replaced with serum autologous to the lymphocyte donor. The ability of subjects' sera to inhibit antibody-dependent cellular cytotoxicity (ADCC) of chicken red blood cells and natural killer (NK) of K562 cells by normal donor lymphocytes was assessed by a $^{51}$Cr (New England Nuclear Corp., Boston, MA, U.S.A.) release assay. Assays were modified from standard methods (Garovoy & Carpenter, 1980). Heat-inactivated test serum was added to a final concentration of 11%, and supernatants collected with a Skatron filter collection system (Flow Laboratories, Hamden, CT, U.S.A.). Inhibition was defined as test CPM release less than the control release minus 2 s.d. PHA-induced blast cells from inhibited effectors were used as potential ADCC targets to detect antibodies against histocompatibility antigens. Serum inhibition of E rosettes was assessed by preincubating normal donor lymphocytes for 1 h at 4°C, washing, and testing the cells for rosette formation with washed sheep red blood cells. Typing for HLA antigens was kindly provided by Dr Paul Terasaki. Skin tests for delayed hypersensitivity were also performed with PPD, SK-SD, and mumps antigens. Statistical analysis was performed as previously reported (Schuelke *et al.*, 1982).

Nineteen individuals represented in Table I and the Figure had intrinsically low blastogenic responses to one or more mitogen when cultured in media containing FCS. Three (III-9, III-29, IV-14) of the 4 living cancer-patients (III-9, III-29, III-38, IV-14) had lowered lymphocyte activity several years after cancer surgery. Benign breast disease in Individual III-39 and advanced age (74 years) in Individual

TABLE I.—*Subjects whose lymphocytes gave responses below the normal range with one or more mitogen in FCS-containing culture medium*

| | | Mitogen | | |
|---|---|---|---|---|
| Subject | Medium alone | Con-A[2] | PHA[3] | PWM[4] |
| II-15 | 72 ± 45[1] | 4439 ± 267 | 14366 ± 601 | 3103 ± 376 |
| III-6 | 54 ± 38 | 17336 ± 1143 | ND[5] | 4543 ± 670 |
| III-7 | 39 ± 19 | 12608 ± 231 | 17509 ± 1154 | 2930 ± 126 |
| III-8 | 78 ± 22 | 15508 ± 3712 | 14926 ± 1578 | 2417 ± 272 |
| III-9 | 68 ± 11 | 14344 ± 1167 | 16627 ± 817 | 2785 ± 220 |
| III-10 | 69 ± 61 | 23548 ± 1760 | 25225 ± 6021 | 3402 ± 114 |
| III-12 | 93 ± 60 | 10466 ± 857 | 5321 ± 366 | 11293 ± 1178 |
| III-16 | 99 ± 73 | 23059 ± 1713 | 22757 ± 815 | 3411 ± 71 |
| III-17 | 10 ± 8 | 25964 ± 2619 | 30917 ± 4115 | 3149 ± 218 |
| III-22 | 145 ± 13 | 4524 ± 1365 | 16509 ± 6477 | 3482 ± 221 |
| III-29 | 235 ± 168 | 21436 ± 1872 | 14588 ± 2041 | 14383 ± 254 |
| III-31 | 101 ± 13 | 40325 ± 242 | 13110 ± 2410 | 14951 ± 1584 |
| III-33 | 82 ± 63 | 24959 ± 1024 | 45895 ± 2645 | 3605 ± 287 |
| III-34 | 43 ± 1079 | 16143 ± 1079 | 8603 ± 762 | 13213 ± 2308 |
| III-39 | 53 ± 32 | 20560 ± 876 | 16045 ± 1034 | 11666 ± 1105 |
| IV-3 | 117 ± 100 | 36932 ± 5498 | 42160 ± 1474 | 2671 ± 142 |
| IV-9 | 41 ± 12 | 38396 ± 2995 | 32648 ± 3811 | 3168 ± 106 |
| IV-14 | 141 ± 114 | 35936 ± 2697 | 23262 ± 1087 | 3184 ± 228 |

1—Numbers represent mean CPM ± 1 s.d.

2—Normal 95% range = $2{\cdot}675 \times 10^4$–$5{\cdot}166 \times 10^4$ CPM.

3—Normal 95% range = $1{\cdot}866 \times 10^4$–$4{\cdot}929 \times 10^4$ CPM.

4—Normal 95% range = $4{\cdot}07 \times 10^3$–$3{\cdot}868 \times 10^4$.

5—Culture lost due to microbial contamination.

FIGURE

1 2 Code number

Male or female unaffected

Cancer verified by pathology

Multiple primary cancers by family history

Cancer by family history

Proband

Individuals who were Immunologically tested

Individuals with serum IgM > 420 mg/dl

Ov 50 Cancer site and age at diagnosis d 49 Age at death

63 Age at time of study

(50) mg/dl of serum IgA in those individuals found to be below the 95% range

Sk Skin

Ov Ovary

CSU Cancer site unknown

Ut Uterus

Le Leukemia

K Kidney

GB Gallbladder

Pr Prostate

Bl Urinary bladder

Lu Lung

Co Colon

B Breast

Lym Lymphoma

OS Osteogenic Sarcoma

C Low intrinsic Con-A response

P Low intrinsic PHA response

W Low intrinsic PWM response

SC Autologous serum inhibited Con-A response

SP Autologous serum inhibited PHA response

SW Autologous serum inhibited PWM response

NK Serum inhibited NK killing

AD Serum inhibited ADCC killing

E Serum inhibited E rosette formation

CF 013
I
II
III
IV

TABLE II.—*Subjects whose sera decreased the blastogenic response of autologous lymphocytes to one or more mitogens*

| | Serum | | | | | | | |
|---|---|---|---|---|---|---|---|---|
| | FCS[1] | | | | Autologous[1] | | | |
| Subject | Medium alone | Con-A[2] | PWM | PHA | Medium alone | Con-A | PWM | PHA |
| III-9 | 68 ± 11 | 14344 ± 1167 | 2785 ± 220 | 16627 ± 817 | 126 ± 46 | 60 ± 18 | 1432 ± 115 | 1840 ± 66 |
| III-20 | 26 ± 25 | 25982 ± 1772 | 3776 ± 38 | 20885 ± 1633 | 71 ± 61 | 9702 ± 884 | 4276 ± 117 | 29771 ± 897 |
| III-28 | 68 ± 30 | 54838 ± 1553 | 18998 ± 1187 | 47116 ± 2111 | 274 ± 198 | 56813 ± 3698 | 66866 ± 646 | 27548 ± 2873 |
| III-36 | 36 ± 22 | 50266 ± 7092 | 6681 ± 373 | 66527 ± 2235 | 74 ± 56 | 26359 ± 3269 | 5999 ± 287 | 64301 ± 2489 |
| III-39 | 53 ± 32 | 20560 ± 976 | 11666 ± 1105 | 16045 ± 1034 | 51 ± 44 | 71 ± 23 | 2303 ± 175 | 1410 ± 302 |
| III-48 | 54 ± 32 | 83003 ± 3811 | 24478 ± 2917 | 34658 ± 6977 | 95 ± 43 | 62617 ± 1305 | 52021 ± 2996 | 36349 ± 3652 |
| III-50 | 93 ± 25 | 35346 ± 688 | 7772 ± 718 | 46428 ± 2145 | 276 ± 94 | 19181 ± 3297 | 10779 ± 1108 | 53285 ± 4726 |
| IV-9 | 41 ± 12 | 38396 ± 2995 | 3168 ± 106 | 32648 ± 3811 | 134 ± 103 | 17165 ± 1889 | 4020 ± 270 | 49743 ± 2687 |
| IV-13 | 105 ± 35 | 52107 ± 4988 | 14315 ± 1087 | 56488 ± 5504 | 68 ± 44 | 49605 ± 721 | 26813 ± 1533 | 38309 ± 3136 |
| IV-17 | 65 ± 15 | 37787 ± 4658 | 12595 ± 1400 | 30891 ± 2205 | 346 ± 312 | 29029 ± 2121 | 30425 ± 1153 | 19744 ± 3800 |
| IV-22 | 82 ± 17 | 63502 ± 1019 | 27715 ± 2120 | ND | 346 ± 213 | 52418 ± 2815 | 72206 ± 1835 | ND |
| IV-35 | 30 ± 13 | 70988 ± 4232 | 18875 ± 4232 | 58595 ± 1865 | 13 ± 2 | 30223 ± 1125 | 22910 ± 3750 | 53267 ± 1683 |

1–Lymphocytes stimulated in media containing either FCS or autologous serum.

2–Mean CPM ± 1 s.d. of cultures stimulated with the mitogen indicated. Normal ranges as for Table I.

ND–Not determined.

II-15 were the only other factors identified which might explain the other low responses.

The probabilities of randomly selecting a population with the observed number or a more extreme number of individuals below the normal ranges were calculated to be $P \simeq 0{\cdot}001$, $P \simeq 0{\cdot}014$ and $P \simeq 2 \times 10^{-8}$ for Con-A, PHA, and PWM respectively.

Table II and the Figure show that sera from 12 subjects caused a lower blastogenic response to one or more mitogen, even though the most common observation was unchanged or increased responsiveness. This suppressive activity was evident in one cancer patient (III-9), whose serum suppressed the responses of all 3 mitogens. Inhibitory activity(ies) were also present in 2 spouses (III-39, III-50). The most common selective suppression was of Con-A responses (III-36, III-48, III-50, IV-9, IV-22, IV-35), followed by PHA (III-20, III-28, IV-17), while selective PWM suppression was not observed. A sex-influenced trend for females preferentially to express serum-mediated mitogenic suppression is suggested by the fact that only 3 men (III-9, III-20, III-50) showed such activity, although the sex ratio of the family members sampled was approximately equal.

TABLE III.—*Inhibition of NK effector by family sera**

| Serum | %Maximal $Cr^{51}$ release† |
|---|---|
| Control | $56{\cdot}9 \pm 2{\cdot}6$ |
| III-4 | $46{\cdot}5 \pm 0{\cdot}7$ |
| III-5 | $42{\cdot}9 \pm 2{\cdot}0$ |
| III-10 | $43{\cdot}5 \pm 2{\cdot}7$ |
| III-17 | $40{\cdot}2 \pm 0{\cdot}1$ |
| III-22 | $40{\cdot}8 \pm 1{\cdot}8$ |
| III-29 | $44{\cdot}7 \pm 0{\cdot}5$ |
| III-33 | $43{\cdot}5 \pm 3{\cdot}0$ |
| III-35 | $43{\cdot}7 \pm 1{\cdot}0$ |
| III-39 | $49{\cdot}4 \pm 0{\cdot}1$ |
| IV-11 | $47{\cdot}6 \pm 1{\cdot}3$ |
| IV-13 | $39{\cdot}8 \pm 2{\cdot}0$ |
| IV-14 | $44{\cdot}3 \pm 1{\cdot}7$ |
| IV-36 | $42{\cdot}4 \pm 1{\cdot}7$ |

* Only the data on inhibitory sera are presented. The other sera did not exceed control values by 2 s.d.

† Data presented as average % release ± s.d. Percentage release was calculated by the formula:

$$\% \text{ release} = \frac{ER - SR}{MR - SR} \times 100$$

Where ER = experimental release, MR = maximal release, and SR = spontaneous release.

Serum-mediated inhibition of NK activity (Table III and the Figure) was presented in 11 bloodline individuals and 2 spouses (III-10, III-39). NK inhibitory activity occurred both concomitantly with and independently of ADCC suppressor activity, and was present in the 2 cancer patients with lower serum IgA (III-29, IV-14). Sera from 2 apparently well generation IV females (IV-13, IV-36) also inhibited NK activity.

The Figure shows the 8 bloodline individuals (III-5, III-8, III-17, III-20, III-22, III-52, IV-9, IV-32) whose sera inhibited ADCC effector activity. None of these sera had demonstrable antibodies against the effector cells. Inhibitory activity was present in 2 younger generation IV women (IV-9, IV-32). Based on the results of sera samples from 300 midwestern residents without a known ovarian cancer propensity in their families, it was predicted that 1 in 50 sera would be inhibitory. Thus the probability of randomly selecting a population with 8 or more out of 50 individuals having the activity is calculated to be $P \simeq 3{\cdot}2 \times 10^{-4}$ (Fisher's exact test).

Three sera inhibited E-rosette formation by normal donor T lymphocytes (III-9, III-39, IV-14). Two sera were from cancer-affected individuals (III-9, IV-14) and one was from a spouse (III-39) subsequently proven to have benign breast disease yet who underwent mastectomy. Direct assessment of E-rosette formation by subjects' lymphocytes in the absence of serum failed to reveal any abnormalities not attributable to noncancerous conditions.

No simple association was evident between any single significant abnormal immune finding and blood markers (ABO, Duffy, HLA, or Rhesus antigens) present in the kindred. Skin test results were unremarkable.

Any close association between an immunological parameter (or parameters) and a cancer-prone genotype would aid immeasurably in genetic counselling and decision logic for surveillance and management. Long-term follow-up will obviously be needed to determine if any immune parameter(s) identified in cancer families are a constitutionally integral trait correlating with cancer-predisposing genotype and eventual phenotypic expression (clinical cancer).

The immune findings in spouses (III-10, III-12, III-39 and III-50) could represent either random findings unrelated to cancer or a communicable environmental effect (Byers *et al.*, 1975; Dean *et al.*, 1979; Dworsky *et al.*, 1978; Graham-Pole *et al.*, 1976; Guirgis *et al.*, 1978; Hersey *et al.*, 1979). The following observations are consistent with an association of immune findings with cancer-prone genotype: (1) predominance of mitogen suppressive activity in sera from high-risk females; (2) instances of selective mitogen inhibition by family sera similar to hepatocellular carcinoma sera effects on mitogen responses (Ren & Chan, 1981) and (3) increased frequency of ADCC and/or NK serum inhibitory activity in older generation III individuals and selective inhibition of one killing activity by some sera is strikingly similar to the results of a mouse tumour study (Nair *et al.*, 1980). Limitations in sample size and the findings in a single family restrict a more complete elucidation of immunological mechanisms. Logistic and other considerations have to date prevented more extensive immunological analysis than the assays performed as reported above. However, we feel that these data are sufficiently suggestive to warrant further investigations of immune function in other ovarian cancer-prone kindreds in an attempt to identify both a precancerous marker (or markers) and to define further possible aetiological relationships between immune functions and hereditary cancer.

Support for this work was provided (in part) by NIH Grant No. RR05390, the Elsa U. Pardee Foundation, and the Fraternal Order of Eagles.

We wish to thank Diane Stanley for her assistance in the typing and assembly of this manuscript, Brenda Novak, for her expert technical assistance, and Pamela R. Fain, for her critical manuscript evaluation.

### REFERENCES

BADGER, A. M., OH, S. K. & MOOLTEN, F. (1981) Differential effects of an immunosuppressive fraction from ascites fluid of patients with ovarian cancer on spontaneous and antibody-dependent cytotoxicity. *Cancer Res.*, **41**, 1133.

BYERS, V. S., LEVIN, A. S., HACKETT, A. J. & FUDENBERG, H. H. (1975) Tumor-specific cell-mediated immunity in household contacts of cancer patients. *J. Clin. Invest.*, **55**, 500.

CHAPERON, E. A. (1982) Suppression of lymphocytes by cephalosporins. In *The Influence of Antibiotics on the Host–Parasite Relationship.* (Eds Eickenberg, *et al.*) Berlin: Springer-Verlag. p. 22.

DAUNTER, B., KHOO, S. K. & MACKAY, E. V. (1979) Lymphocyte response to plant mitogens. I. The distribution of lymphocyte response to various doses of phytohemagglutinin-M in pregnant women and women with carcinoma of the cervix or ovary. *Gynecol. Oncol.*, **7**, 309.

DEAN, J. H., GREENE, M. H., REIMBER, R. R. & 6 others (1979) Immunologic abnormalities in melanoma-prone families. *J. Natl Cancer Inst.*, **63**, 1139.

DWORSKY, R., BAPTISTA, J., PARKER, J. & 5 others (1978) Immune function in healthy relatives of patients with malignant disease. *J. Natl Cancer Inst.*, **60**, 27.

GAROVOY, M. R. & CARPENTER, C. B. (1980) Lymphocyte-mediated cytotoxicity. In *Manual of Clinical Immunology.* (Eds Rose & Friedman) Washington: American Society for Microbiology. p. 290.

GERBER, M. A., KOFFLER, D. & COHEN, C. J. (1977) Circulating antibodies in patients with ovarian carcinoma. *Gynecol Oncol.*, **5**, 228.

GRAHAM-POLE, J., OGG, L. T., ROSS, C. E. & COCHRAN, A. J. (1976) Sensitization of neuroblastoma patients and related and unrelated contacts to neuroblastoma extracts. *Lancet*, i, 1376.

GUIRGIS, H. A., LYNCH, H. T., HARRIS, R. E. & VANDEVOORDE (1978) Genetic and communicable effects on carcinoembryonic antigen expressivity in the Cancer Family Syndrome. *Cancer Res.*, **38**, 2523.

HERSEY, P., EDWARDS, A., HONEYMAN, M. & MCCARTHY, W. H. (1979) Low natural killer cell activity in familial melanoma patients and their relatives. *Br. J. Cancer*, **40**, 113.

HESS, A. D., GALL, S. A. & DOWSON, J. R. (1979) Inhibition of *in vitro* lymphocyte function by cystic and ascitic fluids from ovarian cancer patients. *Cancer Res.*, **39**, 2381.

HUGHES, N. R. (1971) Serum concentrations of $\gamma$G, $\gamma$A, and $\gamma$M immunoglobulins in patients with carcinoma, melanoma and sarcoma. *J. Natl Cancer Inst.*, **46**, 1015.

HUMPHREY, L., PANOUSSOPOULOS, D., VOLENEC, F. J., *et al.* (1977) Role of tumor immunity in ovarian cancer. *Scot. Med. J.*, **70**, 1186.

LYNCH, H. T., ALBANO, W., BLACK, L., LYNCH, J., RECABAREN, J. & PIERSON, R. (1981) Familial excess of cancer of the ovary and other anatomic sites. *JAMA*, **245**, 261.

MANDELL, G. L., FISHER, R. I., BOSTICK, F. & YOUNG, R. C. (1979) Ovarian cancer: a solid tumor with evidence of normal cellular immune function but abnormal B cell function. *Am. J. Med.* **66**, 621.

MANTOVANI, A., ALLEVENA, P., SESSA, C., BOLIS, G. & MANGIONI, C. (1980*a*) Natural killer activity of lymphoid cells isolated from human ascitic ovarian tumours. *Int. J. Cancer*, **25**, 573.

MANTOVANI, A., POLENTARUTTI, N., PERI, G. & 4 others (1980*b*) Cytotoxicity on tumor cells of peripheral blood monocytes and tumor-associated macrophages in patients with ascites ovarian tumors. *J. Natl Cancer Inst.*, **64**, 1307.

MIKULSKI, S. M., BILLING, R. & TERASAKI, P. I. (1977) Inhibition of effector cell function in human antibody-dependent cellular cytotoxicity by sera from cancer patients. *J. Natl Cancer Inst.*, **58**, 1485.

NAIR, P. N. M., FERNANDES, G., ONOE, K. & 4 others (1980) Inhibition of effector cell functions in natural killer cell activity (NK) and antibody-dependent cellular cytotoxicity (ADCC) in mice by normal and cancer sera. *Int J. Cancer*, **25**, 667.

PATTILLO, R. A., RUCKERT, A. C. F., STORY, M. T., & MATTINGLY, R. F. (1979*a*) Immunodiagnosis in ovarian cancer: blocking factor activity. *Am. J. Obstet. Gynecol.*, **133**, 791.

PATTILLO, R. A., STORY, M. T. & RUCKERT, A. C. F. (1979*b*) Expression of cell-mediated immunity and blocking factor using a new line of ovarian cancer *in vitro*. *Cancer Res.*, **39**, 1185.

POULTON, T. A., CROWTHER, M. E., HAY, F. C. & NINEHAM, L. J. (1978) Immune complexes in ovarian cancer. *Lancet*, ii, 72.

REN, E. C. & CHAN, S. H. (1981) Inhibition of lymphocyte proliferation by sera from patients with hepatocellular carcinoma: lack of correlation with serum alpha-fetoprotein levels. *J. Natl Cancer Inst.*, **66**, 625.

SCHUELKE, G. S., LYNCH, H. T., LYNCH, J. F., FAIN, P. R. and CHAPERON, E. A. (1982) Low serum IgA in a familial ovarian cancer aggregate. *Cancer Genet. Cytogenet.*, **6**, 231.

UEDA, K., TOYOKAWA, M., NAKAMORI, H. & 5 others (1978) Immunosuppressive effect of serum in patients with ovarian carcinoma. *Obstet. Gynecol.*, **51**, 225.

UEDA, K., TOYOKAWA, M., NAKAMORI, H. & 5 others (1979) The prognostic value of serum immunosuppressive effect in patients with ovarian cancer. *Obstet. Gynecol.*, **53**, 480.